# Genetic diversity of *Plasmodium malariae* in sub-Saharan Africa: a two-marker genotyping approach for molecular epidemiological studies

**DOI:** 10.3389/fcimb.2024.1405198

**Published:** 2024-07-19

**Authors:** Miriam Rodi, Katarzyna Kawecka, Laura Stephan, Lilith Berner, Martha Salinas Medina, Albert Lalremruata, Tamirat Gebru Woldearegai, Pierre Blaise Matsiegui, Mirjam Groger, Rella Zoleko Manego, Dorothea Ekoka Mbassi, Ghyslain Mombo-Ngoma, Selidji Todagbe Agnandji, Michael Ramharter, Benjamin Mordmüller, Juliana Inoue, Jana Held

**Affiliations:** ^1^ Institute of Tropical Medicine Tübingen, University Hospital Tübingen, Tübingen, Germany; ^2^ German Center for Infection Research Deutsches Zentrum für Infektionsforschung (DZIF), partner site Tübingen, Tübingen, Germany; ^3^ Centre de Recherches Médicales de la Ngounié, Fougamou, Gabon; ^4^ Center for Tropical Medicine, Bernhard Nocht Institute for Tropical Medicine & I, Department of Medicine, University Medical Center Hamburg-Eppendorf, Hamburg, Germany; ^5^ German Center for Infection Research Deutsches Zentrum für Infektionsforschung (DZIF), partner sites Hamburg-Lübeck-Borstel-Riems, Hamburg, Germany; ^6^ Centre de Recherches Médicales de Lambaréné (CERMEL), Lambaréné, Gabon; ^7^ Department of Implementation Research & I, University Medical Center Hamburg-Eppendorf, Hamburg, Germany; ^8^ Department of Medicine, University Medical Center Hamburg-Eppendorf, Hamburg, Germany; ^9^ Department of Medical Microbiology, Radboud University Medical Center, Nijmegen, Netherlands

**Keywords:** *Plasmodium malariae*, genotyping, size polymorphism, *pmtrap*, *pmmsp1* F2

## Abstract

**Introduction:**

*Plasmodium malariae* is the most common non-falciparum species in sub-Saharan Africa. Despite this, data on its genetic diversity is scarce. Therefore, we aimed to establish a *P. malariae* genotyping approach based on size polymorphic regions that can be easily applied in molecular epidemiological studies.

**Methods:**

Four potential genotyping markers, Pm02, Pm09, *P. malariae* thrombospondin-related anonymous protein (pmtrap), and *P. malariae* merozoite surface protein fragment 2 (pmmsp1 F2) were amplified via nested PCR and analysed using automated capillary gel electrophoresis.

**Results:**

We observed the highest allelic diversity for pmtrap (MOI = 1.61) and pmmsp1 F2 (He = 0.81). Further applying the two markers pmtrap and pmmsp1 F2 on a different sample set of 21 *P. malariae* positive individuals followed up over one week, we saw a high consistency in their performance. The results show a large complexity and high dynamics of *P. malariae* infections in the asymptomatic Gabonese study population.

**Discussion:**

We successfully implemented a new genotyping panel for *P. malariae* consisting of only two markers: pmtrap and pmmsp1 F2. It can be easily applied in other settings to investigate the genotype diversity of *P. malariae* populations, providing further important data on the molecular epidemiology of this parasite species.

## 1 Introduction

*Plasmodium malariae* is the second most abundant *Plasmodium* species in sub-Saharan Africa causing malaria in humans (Garnham, 1966; Coatney, 1971; Sutherland, 2016; Nundu et al., 2021; Culleton et al., 2023; Miezan et al., 2023). It is detected worldwide in many tropical and subtropical regions and is often found in co-infections with other malaria parasites, mostly *P. falciparum* (Zhou et al., 1998; Herman et al., 2023; Nguiffo-Nguete et al., 2023). Over the past decades a worldwide decline in malaria cases and deaths has been reported by the World Health Organization (WHO) (Organization, 2023). However, this decrease in case numbers has not been observed for *P. malariae*. On the contrary, there are many endemic countries reporting an increase in *P. malariae* prevalence (Cao et al., 2016; Yman et al., 2019; Agonhossou et al., 2022; Nainggolan et al., 2022). Due to improvements and increased usage of molecular diagnostics, the number of *P. malariae* infections has been shown to be substantially higher than previously described (Woldearegai et al., 2019; Mbama Ntabi et al., 2022; Nguiffo-Nguete et al., 2023). However, treatment as well as epidemiological studies and vaccine development are mainly directed towards *P. falciparum* and more recently also *P. vivax*, while other species are almost entirely neglected (Groger et al., 2022). A deeper understanding of the biology and epidemiology of the non-*falciparum* species would contribute to our understanding of transmission dynamics which is a prerequisite for achieving malaria elimination.

*P. malariae* causes a more benign form of malaria characterized by comparably mild symptoms and low parasitemia. However, serious complications have also been reported, mainly including severe anemia (Langford et al., 2015) and acute kidney injury (Ehrich and Eke, 2007). Infections are described as more chronic, persisting in the human host for a long time, indicating that *P. malariae* is successfully adapted to its human host.

A powerful tool to further characterize a parasite population and its transmission dynamics is the usage of genotyping markers. With this approach, diverse genetic regions are used to distinguish parasite genotypes. For *P. malariae* two genotyping marker panels have been identified previously (Bruce et al., 2007; Mathema et al., 2020). Both approaches focus on microsatellite markers, that represent genomic repetitive regions displaying a high genetic diversity. Few other *P. malariae* genes have been investigated additionally regarding their variability (Guimarães et al., 2015; Lalremruata et al., 2017; Srisutham et al., 2018; Putaporntip et al., 2022). In the present study, we aimed to establish a panel with a minimum number of genotyping markers for *P. malariae* yet with a high discriminative power. We assessed four markers from previous studies - two microsatellites, Pm02 and Pm09, as well as *P. malariae* thrombospondin-related anonymous protein (*pmtrap*), and *P. malariae* merozoite surface protein 1 fragment 2 (*pmmsp1* F2) - the most promising candidate markers based on published diversity and successful amplification (Bruce et al., 2007; Srisutham et al., 2018; Mathema et al., 2020). We optimized the workflow, confirmed their diversity in a large set of *P. malariae* samples from Gabon, a malaria-endemic country in sub-Saharan Africa, and analysed dynamics and complexity of infections, by following-up asymptomatically infected individuals from Gabon in a tight schedule over one week.

## 2 Methods

### 2.1 Study populations

A total of 410 *P. malariae* samples from two different study populations were investigated in this study. Study 1 was a cross-sectional study, aiming to characterize *Plasmodium* infections in rural areas in Fougamou and surrounding villages in Gabon in February/March 2016 (Manego et al., 2017; Groger et al., 2018; Woldearegai et al., 2019). A total of 840 individuals aged from 1-96 years were included in the original study, from which 193 participants were positive by 18S rRNA qPCR for *P. malariae.* Of these, 95 samples were used here, selected according to low Cq values.

Study 2 was conducted in 2019/2020 in Lambaréné, Gabon, assessing safety and efficacy of ivermectin against *P. falciparum* infections in asymptomatic adults (Ekoka Mbassi et al., 2023); samples were collected throughout the year. As ivermectin at the given dose showed similar activity as placebo against Plasmodia, individuals are regarded as not treated for this study. Out of 49 participants, 21 were positive for *P. malariae* at inclusion. The following 15 timepoints were investigated: Screening (SCR), after 8 (H8), 16 (H16), 24 (H24), 32 (H32), 40 (H40), 48 (H48), 56 (H56), 64 (H64), 72 (H72), 96 (H96), 120 (H120) hours, day 6 (D6), day 7 (D7). On day 7 a complete treatment of artemether-lumefantrine was given, and additional sampling was done on day 14 (D14).

Both studies and the corresponding experimental protocols were approved by the Institutional Ethics Committee of CERMEL (CEI-007/2014, CEI/CERMEL 006/2019). Study 2 was registered with the Pan-African Clinical Trials Registry (PACTR201908520097051). All methods were carried out in accordance with relevant guidelines and regulations. Informed consent was obtained from all adult participants or legal guardians.

### 2.2 DNA extraction and *Plasmodium* species determination

Blood samples were stabilized in RNAlater prior to extraction. DNA was extracted either manually using the QIAamp DNA mini blood kit (QIAGEN) or automated in the QIAsymphony (QIAGEN) or in the KingFisher™ Flex Purification System (Thermo Fisher Scientific) with the sbeadex blood kit (LGC), according to the manufacturer’s protocols. *Plasmodium* species were determined by qPCR as described previously targeting the 18S rRNA of the different *Plasmodium* species (Groger et al., 2018; Woldearegai et al., 2019; Ekoka Mbassi et al., 2023).

### 2.3 Identification of *P. malariae* genotyping markers

We chose four loci distributed on different regions of the *P. malariae* genome, namely Pm02, Pm09, *pmtrap*, and *pmmsp1* F2, based on high genetic diversity published in other *P. malariae* populations (Bruce et al., 2007; Guimarães et al., 2015; Srisutham et al., 2018). Pm09 is located on chromosome 1 and *pmmsp1* F2 on chromosome 7. Pm02 and *pmtrap* are both located on chromosome 12. The two microsatellite markers show the following repeat sizes: Pm02 has a repeat unit of 4 bp (CATA) and Pm09 of 17 bp (GCAAAATAACAAAAAGA). *Pmtrap* shows different patterns of 12 bp repeat units (CCAGAGGATAGA; CCAGAGAATAGA; CCAGAGAATAGT).

Each of the four length-polymorphic markers was amplified by nested PCR. In all PCR runs the following controls were used: non-template control (H_2_O), negative control (human whole blood from a malaria naïve person), positive control (*P. malariae* mono infection).

Optimized cycling conditions and primer sequences for Pm02, Pm09, *pmtrap* and *pmmsp1* F2 are shown in **Supplementary Table S1**. New outer (Pm02 and Pm09 forward) and inner (*pmmsp1* F2 and pmtrap) primers were designed according to the amplicon length needed for the analysis method used, which is described below. Primer design was based on the *P. malariae* reference genome PmUG01 sequences (LT594622 – LT594635). Samples that were negative in two consecutive runs, were repeated with 5 more cycles, to exclude low DNA content as a source of error.

### 2.4 Analysis and verification of *P. malariae* marker

Before further analysis, the amplicon size and concentration were measured using automated capillary gel electrophoresis (QIAxcel, QIAGEN). Specific conditions and descriptions are listed in **Supplementary Table S2** and **Supplementary File S1**.

Verification of automated capillary gel electrophoresis is described in **Supplementary File S1**.

### 2.5 Analysis of genetic diversity

We assessed the performance and diversity of the *P. malariae* genotyping markers along 95 *P. malariae* positive samples from study 1. Whereas samples from study 2 were used to analyze the performance of the markers over time. The maximum multiplicity of infection (MOI) was counted based on the highest number of alleles per marker in one sample. The mean MOI for each marker was calculated as followed: $\frac{total\;number\;of\;alleles}{number\;of\;positive\;samples}$ . To estimate the allele frequency for each detected allele size, we divided the number of alleles detected by the total number of PCR positive samples. The expected heterozygosity (*H_e_*) was calculated using the following formula:

$H=\frac{N}{\left(N-1\right)}(1-\sum_{i=1}^{k}p_{i}^{2})$ where p corresponds to the i^th^ of k alleles and N to the number of positive samples.

## 3 Results

### 3.1 Comparative performance and validation of four potential *P. malariae* genotyping markers

Based on literature and *in silico* analysis, we chose four different loci Pm02, Pm09, *pmtrap*, and *pmmsp1* F2.

#### 3.1.1 High diversity of *P. malariae* genotypes in study population from Gabon

**Figure 1:**
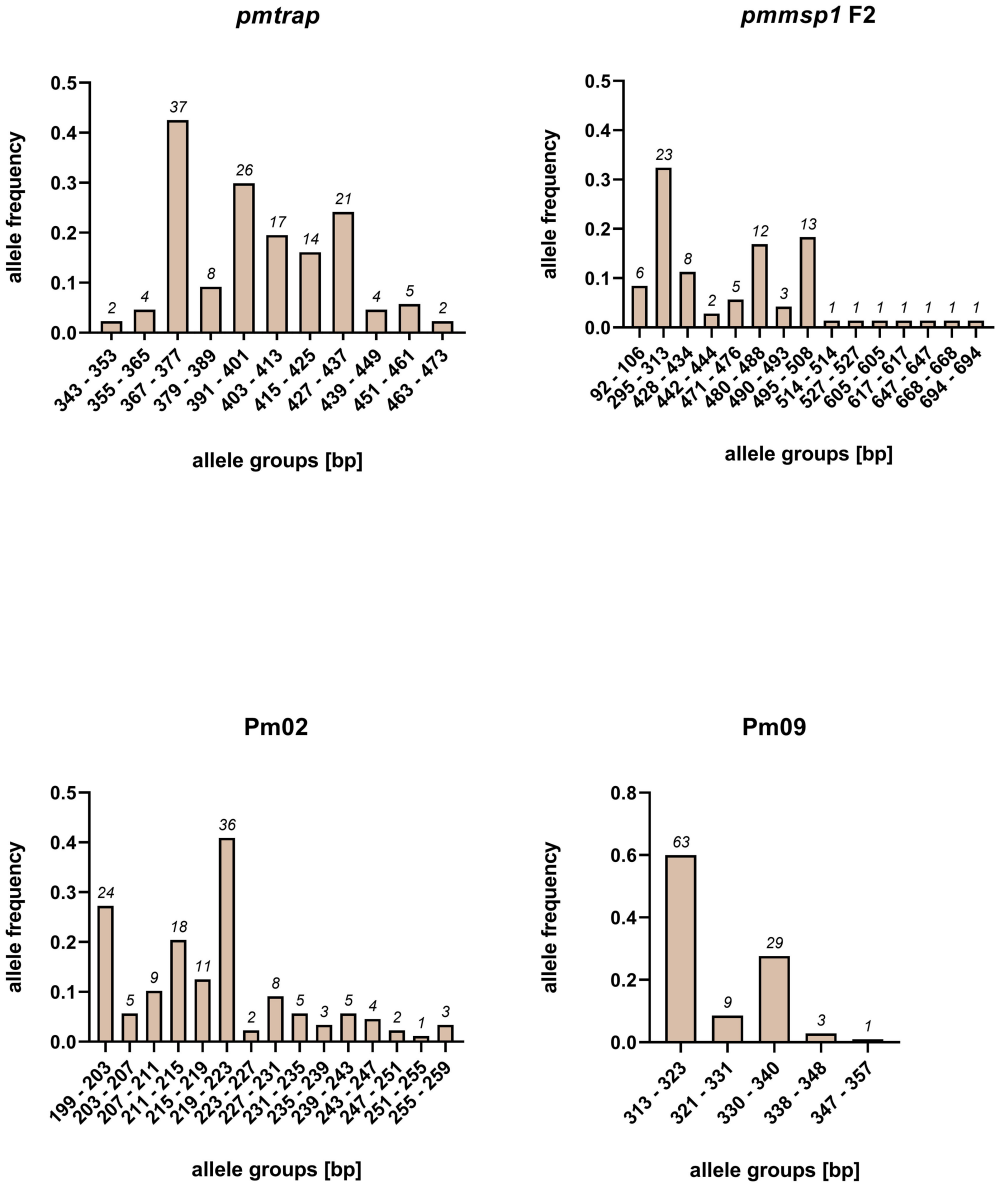
Allele frequencies of four *P. malariae* markers *pmtrap*, *pmmsp1* F2, Pm02 and Pm09, arranged in different allele groups according to the fragment size. Frequencies and sizes include alleles from multiclonal infections. In total 95 samples were screened.

The allelic diversity of the markers was assessed on a set of 95 samples from study 1. The number of allele groups detected in the 95 samples was highest for *pmmsp1* F2 and Pm02, with 15 distinct groups detected each (**Figure 1**). While 11 allele groups were detected for *pmtrap*, Pm09 resulted in 5 distinct allele groups, which was the lowest number detected. Overall, the allele frequency of groups was unevenly distributed for all four markers, with mostly one major allele group. For *pmmsp1* F2 seven minor allele groups were identified, each appearing only once.

An overall comparison of allele characteristics showed that *pmtrap* and *pmmsp1* F2 performed best among the four markers when aiming at displaying diversity (**Table 1**). The highest expected heterozygosity (H_e_) of 0.81 was calculated for *pmmsp1* F2 while *pmtrap* had the highest mean MOI of 1.61. Pm02 performed comparably well, with a mean MOI of 1.55 and an H_e_ of 0.68. Despite the high resolution of the chosen QIAxcel method, the differentiation of the allele groups was challenging for the marker Pm02, as it has a repeat size unit of only 4 bp, which falls within the maximum resolution of 3-5 bp that can be achieved with the underlying method. Pm09 was the least diverse locus, with the lowest H_e_ (0.56) as well as a low MOI (1.19). Amplification success exceeded 90% for three out of the four markers, while *pmmsp1* F2 exhibited a lower performance, with a PCR positivity rate of only 76%. The gene is known for its polymorphisms; therefore, it is likely that for some of the samples the primer regions were too diverse to anneal without mismatches. Indeed, we found one mismatch in the reverse primer region of 1/12 generated *pmmsp1* F2 sequences. We do not see any effect of the Cq value on the amplification success of the samples.

**Table 1:** Allele characteristics of four *P. malariae* markers based on size polymorphisms.

Marker	*P. malariae* positivity by PCR	Number of alleles		MOI		H_e_	Allele size range [bp]
		distinct	total	max.	mean		
*pmtrap*	87/95 [92%]	11	140	6	1.61	0.60	349 – 469
*pmmsp1* F2	72/95 [76%]	15	80	3	1.11	0.81	92 – 694
Pm02	88/95 [93%]	15	136	7	1.55	0.66	200 – 257
Pm09	88/95 [93%]	5	105	4	1.19	0.56	314 - 346

MOI, Multiplicity of infection. H_e_, expected Heterozygosity.

#### 3.1.2 *In silico* analysis reveals new *P. malariae* genotypes from African samples

In addition to the *P. malariae* reference genome obtained from Uganda, we identified sequences from regions outside of Africa within the NCBI database that align to the four markers explored with samples from study 1. We also considered *P. brasilianum* sequences. 32 sequences were retrieved for *pmtrap* and 50 sequences for *pmmsp1*. All 32 *pmtrap* sequences are derived from samples collected in Southeast Asia. 35/50 *pmmsp1* sequences originate from Southeast Asia as well and the remaining 15/50 from South America. For Pm02 and Pm09, one and two published sequences respectively aligned to our sequences, all three originating from South America.

When comparing the sequences from *pmtrap* and *pmmsp1* F2 to the above-mentioned sequences, we found four new genotypes using *pmtrap* (accession numbers: OR829576-OR829579) and five new genotypes using *pmmsp1* F2 (accession numbers: OR829569-OR829571, OR829572, OR829573), among our samples that were not published before. For Pm09 two new genotypes (accession numbers: OR829566, OR829568) were found, each displaying a new repeat unit motif of 17 bp (GAAGAGCAAAATAACAA) and 8 bp (AACAAACA). For Pm02 seven new genotypes were found (accession numbers: OR829554, OR829557, OR829558, OR829560-OR829564), one with a new repeat unit of 6 bp (CCACAC). New genotypes are defined by a new number of repeat units for *pmtrap*, Pm02 and Pm09 or an amplicon size not reported before for *pmmsp1* F2. The genotype derived from the reference genome was found among our sequences in all four markers.

#### 3.1.3 Combination of *P. malariae* genotyping marker reveals high number of samples with one genotype

We investigated which combination of the four markers gives the highest diversity compared to using a single marker (**Supplementary Figure S1**). This was assessed on the 95 samples from study 1. Hereby the number of samples with either 1 (i.e., MOI=1) or more than 1 genotype (i.e., MOI>1), was used as a benchmark to evaluate the different combinations. The more samples with an MOI>1, the better the marker combination reflects the allelic diversity of the sample set. In addition, the PCR failure rate, i.e., number of samples that were not amplified, was used for comparison. The number of negative samples was highest (at least 7/95 samples) when the markers were used separately than in any other combination (less than 6/95 samples). All combinations of two markers enhanced diversity by either reducing the number of negative samples or increasing the number of samples with multiple genotypes.

**Figure 2:**
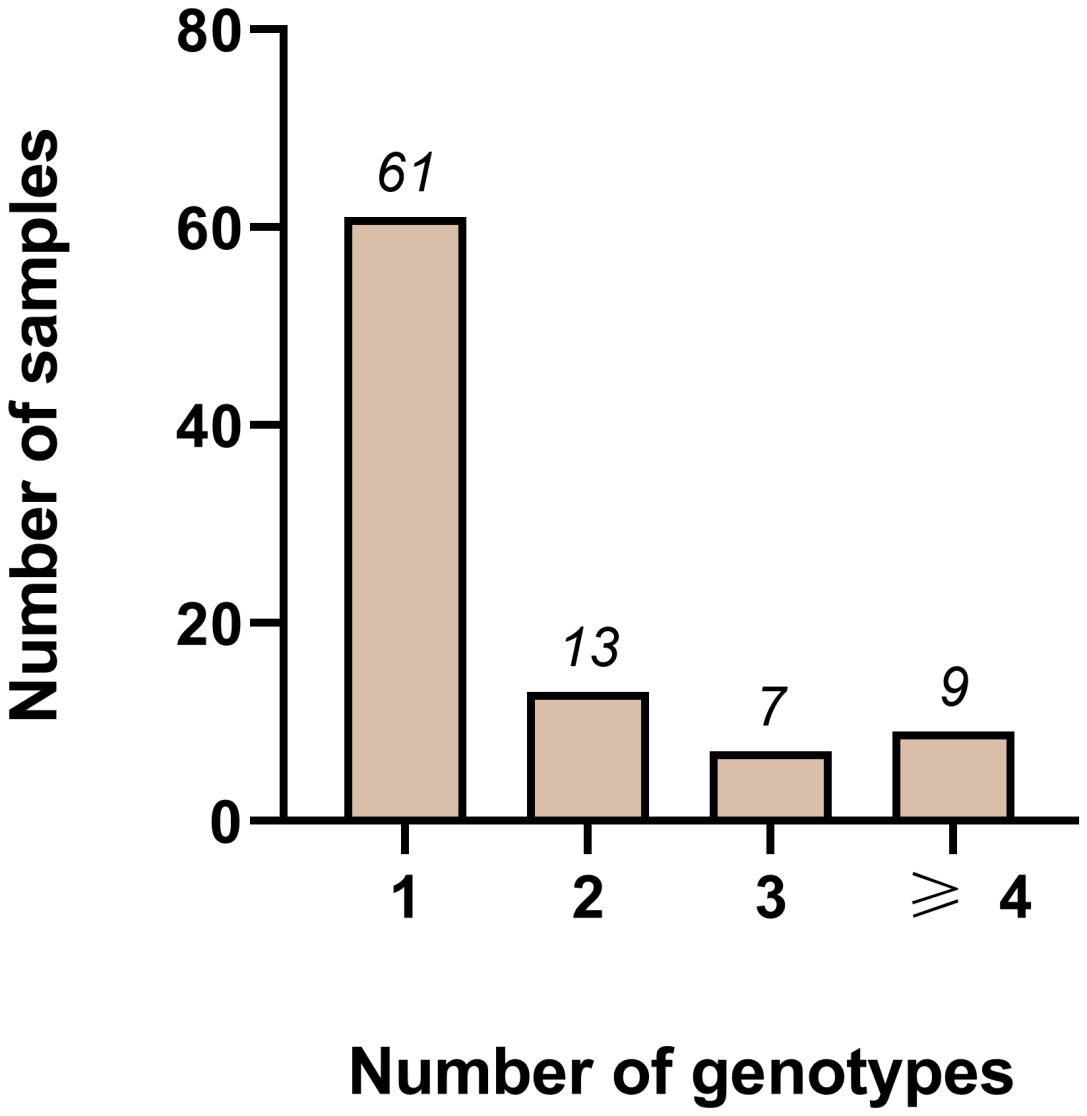
Distribution of different genotypes measured using the two markers *pmmsp1* F2 and *pmtrap* in combination among 95 samples from Gabon. Out of 95 samples, 61 were infected with one genotype only. Whereas 13, 7 and 9 samples were infected with two, three or more than three genotypes respectively. In total 5/95 samples failed to amplify.

For any combination of 3 markers, the multiplicity is even higher, with at least 33/95 samples showing a MOI>1. Combining all four markers decreased the number of negative samples to 3/95 and increased samples with an MOI>1 to 40/95. Although this combination reflects diversity best, we did not select it for further investigation. We excluded Pm09 from the combinations because of its low heterozygosity. Pm02 on the other hand was diverse enough, however the analysis of the generated amplicons was challenging and time consuming as of the small repeat size of 4 bp. Instead, we chose the combination of *pmtrap* and *pmmsp1* F2, identifying 61/95 samples with one genotype only (**Figure 2**). While 5/95 samples were not amplified by any of the two marker, 29/95 samples were carrying two or more genotypes simultaneously.

### 3.2 Indel-based markers *pmmsp1* F2 and *pmtrap* show high consistency in their performance and reveal a large complexity of infection in asymptomatic Gabonese individuals

We further applied the two markers (*pmmsp1* F2 and *pmtrap)* on a unique sample set from study 2, consisting of 21 P*. malariae* positive individuals that were followed for 14 days. In total, 15/21 individuals were positive for both markers by PCR while 6/21 were positive for only one marker respectively (two for *pmtrap* and four for *pmmsp1* F2).

**Figure 3:**
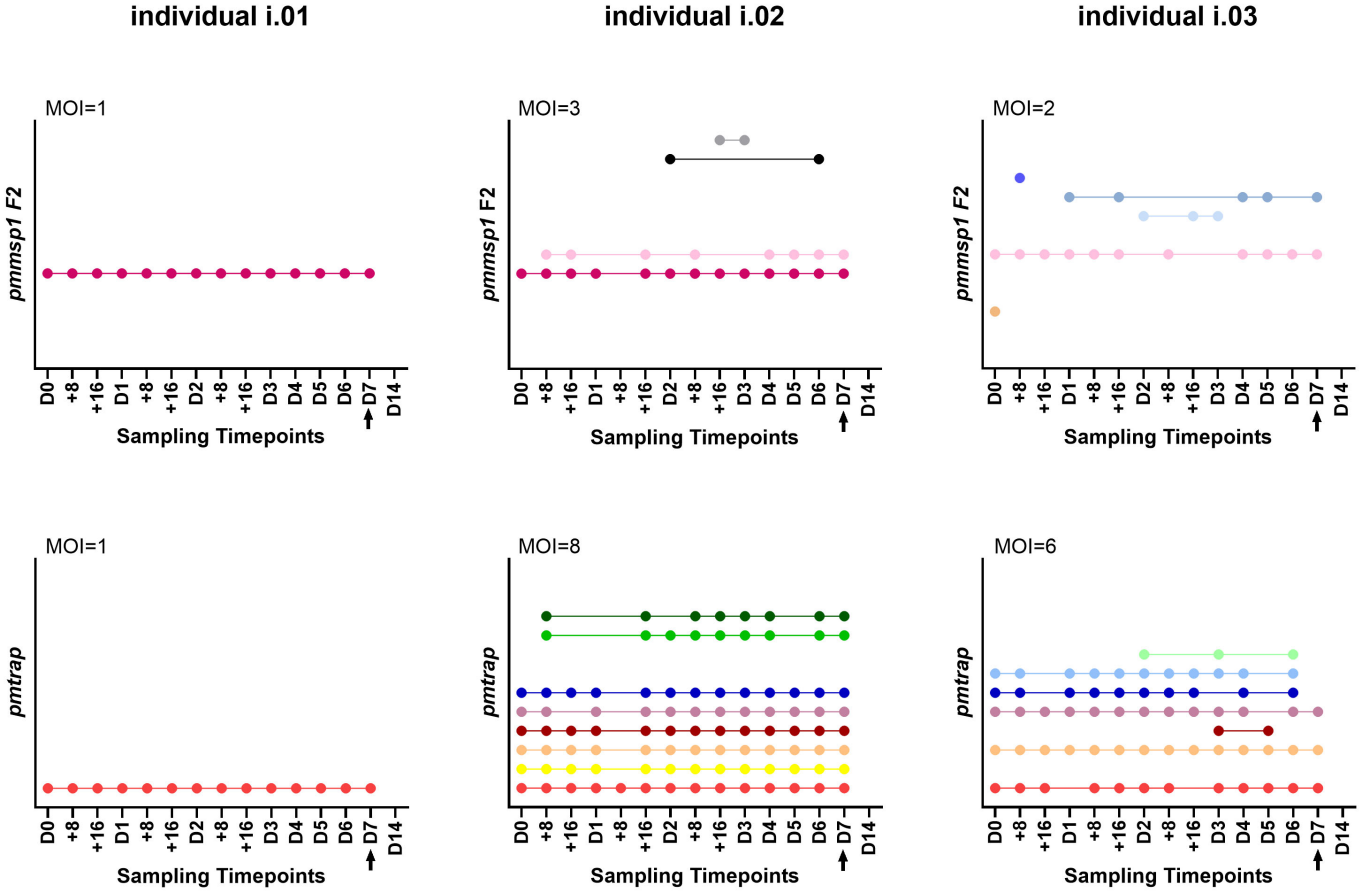
Three individuals i.01, i.02 and i.03 were genotyped at 15 timepoints using the markers *pmmsp1* F2 (upper row) and *pmtrap* (lower row). Each color represents a different genotype for the respective marker. The arrow indicates treatment with artemether-lumefantrine on day 7. The remaining 18 individuals are shown in **Supplementary Figure S2**. MOI, multiplicity of infection, referring to the maximum MOI measured among the 15 timepoints.

Overall individuals showed different patterns throughout the course of the infection (for an overview of all 21 individuals see **Supplementary Table S3**): 2/15 individuals showed only one genotype for both markers over all sampling timepoints (individual i.01 in **Figure 3**) and 11/15 individuals were infected with multiple genotypes according to both markers, one was even detected with 8 genotypes using *pmtrap* (individual i.02 in **Figure 3**). In all 11 mentioned individuals the maximum MOI was higher for *pmtrap* than *pmmsp1* F2. The maximum MOI measured with *pmtrap* and *pmmsp1* F2 is depicted in **Figure 3** for individuals i.01 – i.03 as an example for different genotype patterns detected. The remaining individuals can be seen in **Supplementary Figure S2** (individuals i.04 – i.15). In 2/15 cases the maximum MOI was higher for *pmmsp1* F2 and in two other cases it was similar. Overall, the different genotypes were detected very stable along the 15 timepoints. Some genotypes were fluctuating over time, detected at some timepoints and undetected in others (individual i.03 in **Figure 3**). This shows the high dynamics and complexity of *P. malariae* infections. 17/21 individuals were negative on day 14 (D14), as per protocol participants were treated with artemether-lumefantrine latest on day 7.

## 4 Discussion

The number of *P. malariae* infections in endemic countries has been shown to be substantially higher than previously acknowledged. Nevertheless, there are many open questions regarding the genetic epidemiology of this species. In the present study, we assessed four genotyping markers and selected two of them for establishment of a genotyping panel for *P. malariae* with a minimum number of markers and a high discriminative power.

First, we analysed the individual performance of the four markers using a sample set of 95 *P. malariae* infected individuals from Gabon. Our analysis showed that *pmtrap*, *pmmsp1* F2 and Pm02 are the most diverse among the chosen loci in the population studied. They showed the highest number of distinct alleles - 11, 15 and 15, respectively -, whereas only 5 distinct alleles were found with Pm09 in the 95 samples. The mean MOI was highest for *pmtrap* (1.61), showing that this marker can very well reflect the multiplicity of *P. malariae* infections. The expected heterozygosity was highest for *pmmsp1* F2 (0.81), demonstrating that this marker can detect a wide variety of different *P. malariae* genotypes. In terms of theses parameters, Pm02 performs comparatively well. However, this marker seems unsuitable for the fragment analysis using automated capillary gel electrophoresis with a resolution of 3-5 bp. The repeat size of only 4 bp is too small to reliably distinguish single alleles in infections with multiple genotypes, a limitation of this method. Similar results were found in another study on *P. vivax* genotyping, where automated capillary gel electrophoresis also reached its limit in distinguishing alleles that are close to each other in size (Manrique et al., 2015). The observed differences in the respective PCR efficiency of each marker can be attributed to various influencing factors. Low DNA concentrations in the sample are most likely the major reason why some samples were not amplified at all. We found a mismatch in the primer region in one of the *pmmsp1* F2 sequences. This further underlines the high rate of polymorphisms in this region and might have led to the low amplification success for this marker. If primer binding is not efficient, less DNA is amplified. PCR conditions were thoroughly optimized to increase PCR efficiency to a maximum.

Overall, the range of heterozygosity (0.56 – 0.80) is comparable to other published marker panels by Bruce et al. (0.331 – 0.839) and Mathema et al. (0.530 – 0.922); the mean MOI for the four markers (1.11 – 1.61) is even slightly higher (Bruce et al., 2007; Mathema et al., 2020). However, both parameters depend on the characteristics of the investigated population, as geographical origin, clinical status, participants age, or seasonality. The samples used here for the validation of genetic diversity are from one geographical region (Lambaréné and surrounding villages, Gabon) and were collected within two months (study 1). The same is true for the *P. malariae* sample set from Myanmar of Mathema et al. In contrast Bruce et al. tested samples from diverse geographical origin (Thailand, Malawi, and Gambia), different clinical status and transmission seasons. Therefore, a direct comparison of the different marker panels using different settings can be misleading.

A notable observation for the *pmmsp1* F2 marker was that comparatively more amplicons with a small size were detected and that the MOI was lower than for the other three markers. This effect of preferential amplification of shorter over longer amplicons has been seen in *P. falciparum* genotyping before, targeting the *msp1* locus as well (Messerli et al., 2017). It can lead to an underestimation of the MOI. However, we saw no evidence of preferential amplification of smaller fragments for the *pmmsp1* F2 locus (**Supplementary File S2**). Also, it is unlikely that a low parasite density of the minor genotype leads to these findings in infections with multiple genotypes, as the other three markers have a higher MOI. Another explanation for the seen effect could be mutations in the primer region of the bigger alleles.

We optimized our genotyping approach based on automated capillary gel electrophoresis. To validate the method, we sequenced amplicons from samples that were infected with one genotype only and compared the amplicon lengths from both methods. The high concordance observed for all four markers shows that our chosen analysis method is very reliable (**Supplementary File S3**). This approach has been proven efficient in previous *Plasmodium* genotyping approaches as well (Manrique et al., 2015; Nguyen et al., 2019; Tadele et al., 2022). General advantages of the method include the fast and high throughput, as well as the straightforward analysis. The high deviation of up to a maximum of 21 bp seen for *pmmsp1* F2 can be explained by the lower resolution of 20 bp given by the used method. As the allele size range was bigger for this marker (92 – 694 bp), a method with a lower resolution (20 bp) had to be used.

Previous studies on *P. malariae* genotyping have been carried out in samples from South America and/or Southeast Asia (Guimarães et al., 2015; Srisutham et al., 2018; Putaporntip et al., 2022). The *pmtrap* and *pmmsp1* sequences generated here originate from Gabon and therefore represent a valuable set of genotypes from Africa. A comparison of spatial dynamics of *P. malariae* genotypes reveals that some genotypes appear in all three continents mentioned: for *pmtrap* 3/10 identified genotypes have been found in Southeast Asia as well as Africa; for *pmmsp1* F2 4/25 genotypes have been identified either in Southeast Asia and South America (2 genotypes), in South America and Africa (1 genotype) or in all three continents (1 genotype). However, most genotypes are shared among samples from the same region, forming spatial genotype clusters. As most of the available sequences are from Southeast Asia, the biggest cluster was identified from this region for both markers (4/10 using *pmtrap* and 14/25 using *pmmsp1* F2). Another genotype cluster exclusively seen in samples from South America, consists of three genotypes and was identified using *pmmsp1* F2. We found three new genotypes using both markers in our samples from Gabon, Africa, that were not seen in the sequencing data from any other continent before.

In the end, a combination of the two markers *pmmsp1* F2 and *pmtrap* resulted in the panel with the best discriminative power. While *pmmsp1* F2 can detect a high variety of diverse genotypes, *pmtrap* is able to best cover the multiplicity of *P. malariae* infections. In combination the two markers provide a powerful tool for studies on genetic diversity of *P. malariae*.

Based on the results from the sample set of the two-weekly follow-up study, both markers demonstrated a very consistent performance over time. Genotypes were detected consistently throughout the different sampling timepoints, which shows the method`s effectiveness. We detected up to 8 genotypes in one individual at the same timepoint. This surprisingly high number of circulating *P. malariae* genotypes in an asymptomatic population is particularly interesting. In a previous study from Gabon including symptomatic individuals, *P. malariae* was the species with the highest number of genotypes (Lalremruata et al., 2017). To which extend these findings are related to the clinical status needs further investigation.

Throughout the follow-up period, fluctuations of genotypes were evident, indicating that not all genotypes are present at every sampling occasion. This observation is likely because the amount of DNA from these minor genotypes approaches the limit of detection, so that they are detectable at one time point but not at the other. However, it is also possible that some of the appearing genotypes are new infections. The 72-hour cycle of *P. malariae* could not be seen in the pattern of detected genotypes from our data.

We followed the *P. malariae* infections for a limited time, providing valuable insights into their dynamics. As *P. malariae* infections are known for their longevity and to persist in the human host for even decades (Ciusa et al., 2022), studying infections over longer periods would provide even more information on the biology of this species. However, the high complexity of asymptomatic *P. malariae* infections with multiple genotypes and variable detection, shown by our findings, makes longitudinal investigations challenging to interpret since a genotype may not be detected on one sampling timepoint but be present in another. This could be eventually addressed with next generation sequencing (NGS) approaches that can increase sensitivity and the probability to detect minor clones, potentially adding further insights into *P. malariae* genotypes, infection dynamics and biology (Gruenberg et al., 2019; Wamae et al., 2022).

Some individuals in our study were still PCR-positive on day 14, seven days after treatment with artemether-lumefantrine. We do not think that these are treatment failures, but rather residual DNA that can still be detected with our highly sensitive PCR. Similar results were also found for *P. falciparum* (Inoue et al., 2024). In addition, no parasites were detected microscopically at those timepoints. However, in order to be certain that these are no treatment failures and that the individuals turn PCR negative later, follow-up samples from later timepoints would have been required.

## 5 Conclusion

We were able to successfully implement a new *P. malariae* genotyping panel consisting of only two markers, *pmtrap* and *pmmsp1* F2. This set of markers will add a valuable tool to characterize the genetic diversity of *P. malariae* infections from different regions and to better elucidate the transmission dynamics of this neglected malarial parasite species. It can be implemented in many settings as its usage is straightforward, having a short turnaround time as only two markers are required.

## Data availability statement

The datasets presented in this study can be found in online repositories. The names of the repository/repositories and accession number(s) can be found below: https://www.ncbi.nlm.nih.gov/genbank/, OR829554-OR829580.

## Ethics statement

The studies involving humans were approved by Institutional Ethics Committee of CERMEL. The studies were conducted in accordance with the local legislation and institutional requirements. Written informed consent for participation in this study was provided by the participants’ legal guardians/next of kin.

## Author contributions

MRo: Investigation, Methodology, Supervision, Validation, Visualization, Writing – original draft, Writing – review & editing. KK: Investigation, Methodology, Validation, Writing – review & editing. LS: Investigation, Writing – review & editing. LB: Investigation, Writing – review & editing. MS: Investigation, Methodology, Writing – review & editing. AL: Methodology, Writing – review & editing. TW: Resources, Writing – review & editing. PB: Resources, Writing – review & editing. MG: Resources, Writing – review & editing. RZ: Resources, Writing – review & editing. DE: Resources, Writing – review & editing. GM-N: Resources, Writing – review & editing. SA: Resources, Writing – review & editing. MRa: Resources, Writing – review & editing. BM: Resources, Writing – review & editing. JI: Methodology, Supervision, Validation, Writing – original draft, Writing – review & editing. JH: Conceptualization, Project Administration, Resources, Supervision, Writing – original draft, Writing – review & editing.
