# Supplementary material for: Genetic diversity of Plasmodium malariae in sub-Saharan Africa: a two-marker genotyping approach for molecular epidemiological studies
Source: Front Cell Infect Microbiol. 2024 Jul 19;14:1405198. doi: 10.3389/fcimb.2024.1405198 (PMC11301411; doi:10.3389/fcimb.2024.1405198)
Supplement: Supplementary file 1 [file DataSheet_1.pdf]

frontiers

Supplementary Material

Genetic Diversity of *Plasmodium malariae* in Sub-Saharan Africa: A Two-Marker Genotyping Approach for Molecular Epidemiological Studies

Supplementary File S1. Detailed description of methodology.

Comparison of automated capillary gel electrophoresis to Sanger sequencing

To confirm the fragment size measured by automated capillary gel electrophoresis, we sequenced samples showing only one genotype in all four markers. The aim was to obtain sequences for all four markers from the same 10 samples. 12 µl of each amplicon were purified with 2 µl of Exo-SAP-IT (ThermoFisher Scientific) and incubated at 37°C for 15 min and 80°C for 15 min. Next, the purified amplicons were diluted in nuclease-free water to 1 ng/µl for amplicons of 150 – 300 bp (Pm02) and to 5 ng/µl for amplicons of 300 – 1000 bp (Pm09, *pmtrap* and *pmmsp1* F2). The respective forward and reverse primers from the nested reactions were included at 5 µM and sent for sequencing to Eurofins Genomics. Sequences were analysed using Geneious Prime software (version 2022.2).

Preferential amplification for *pmmsp1* F2

Preferential amplification is known for size polymorphic regions, where under certain conditions during PCR smaller alleles are amplified preferentially relative to bigger alleles (Walsh et al., 1992). We prepared a mock experiment to analyse if this effect occurs for the *pmmsp1* F2 locus, the locus with the largest size range of our markers. For this we used two samples, one showing one allele at a size of 295 bp and the other one at 479 bp. Each sample was diluted 1:10 for five times, until 1:100 000. The stock and the 5 dilutions from each sample were further mixed with the stock of the respective other samples. Dilution details are listed in Supplementary Table S4. Consequently, all mixtures were amplified according to the *pmmsp1* F2 protocol.

Description of allele grouping and bin size setting for the *P. malariae* genotyping markers *pmtrap, pmmsp1* F2, Pm02 and Pm09.

PCR amplicons were analysed using automated capillary gel electrophoresis (QIAxcel, QIAGEN). To discriminate true peaks from background noise a threshold of 10% of the total peak height was set for all runs, so that minor peaks were excluded from subsequent analysis. As positive control a mono-infected *P. malariae* sample with a known amplicon size determined by Sanger sequencing was used in all runs. The amplicon size of each sample was then corrected according to the measured positive control. Depending on the amplicon size and the QIAxcel method used, different size resolutions are achieved (Supplementary Table S2). Therefore, amplicons within a certain size range were binned into allele groups, according to the resolution and the repeat size of the amplicon (detailed description in Supplementary File S1). For the amplicon size ranges of 200 – 257 bp, 314 – 346 bp and 349 – 469 bp for Pm02, Pm09 and *pmtrap* a size resolution of 3-5 bp is achieved with the

QIAxcel method (0M800). The amplicon size for *pmmsp1* F2 is bigger (92 – 694 bp) and therefore, the QIAxcel method (0M400) had to be applied for this marker, resulting in a lower resolution of 20 bp.

Amplicons measured using the QIAxcel were binned into allele groups for each marker. To set the bin size range, the repeat units of Pm02, Pm09 and *pmtrap* served as a benchmark to determine a specific initial size for each allele group. Due to the maximum resolution of 5 bp, the bin size, was defined to be a maximum of +/- 5 bp around the initial allele size. As the repeat unit for Pm02 is 4 bp, only +/- 2 bp were allowed to define the allele groups. The marker *pmmsp1* F2 displays no repeat units and because of the size the resolution is lower and can only distinguish alleles that differ in at least 20 bp. Therefore, allele groups were grouped based on amplicon sizes determined by Sanger sequencing. Major alleles in samples with multiple alleles were defined as those having the highest peak in the QIAxcel electropherogram, i.e., the highest concentration.

Supplementary File S2. Results for preferential amplification in *pmmsp1* F2.

Since for the *pmmsp1* F2 marker the H_e was high, whereas the mean MOI was comparably low (1.11) and smaller alleles occurred with a higher frequency, we analysed if preferential amplification of small alleles could be the reason for this result. However, we could not observe this in our mixed mock infection experiment of two samples with different allele sizes. The bigger allele could still be successfully amplified even at low concentrations when mixed with the smaller allele sample (Supplementary Table S4). Only at the highest dilution of 1:100 000, both samples did not amplify anymore, probably due to the low amount of DNA.

Supplementary File S3. Results for validation of automated capillary gel electrophoresis and sequencing.

To obtain high quality sequences, we had to sequence more than 10 samples for each of the markers from study 1. In total we generated 22 sequences from Pm02 and Pm09, 19 sequences from *pmtrap* and 12 sequences from *pmmsp1* F2.

Sequences were aligned to the *P. malariae* reference chromosomes (LT594622-37) and amplicon lengths were measured from positions of the respective inner forward to the inner reverse primer. All generated sequences aligned to the *P. malariae* reference genome at the expected sites. The concordance between the amplicon lengths obtained from Sanger sequencing from the 10 samples and the results from the automated capillary gel electrophoresis was very high for all four markers (Supplementary Table S5). For Pm09 and *pmtrap*, consistency was the highest, sizes differed in maximum 6 bp. Pm02 and *pmmsp1* F2 showed higher deviations of maximum 11 and 21 bp respectively. The same PCR products were used for sequencing and automated capillary gel electrophoresis, to rule out possible PCR generated errors. Overall, the sequences confirmed the outcome from the automated capillary gel electrophoresis and the resulting allele groups. This reinforces our intention to use the QIAxcel as an analysis tool for the chosen *P. malariae* genotyping markers.

Supplementary Table S1. PCR primer and cycling conditions for four *P. malariae* markers: *pmmsp1* F2, *pmtrap*, Pm02 and Pm09.

**Target region**	**PCR Primer sequence and cycling conditions**	**Reference**
pmmsp1 F2	*Preamplification*	
	F: 5'- CCATACTATTTAATTGCACTAAAG-3'<br>R: 5'- ACACACATAAGCAGTTTTCAAAAA -3'	(Guimarães et al., 2015)
	95°C 3 min (1x); 98°C 20 sec, 63°C 15 sec, 72°C 30 sec (20x); 72°C 2 min (1x); 10°C ∞	This study
	1X KAPA HiFi buffer; 0.3 mM dNTPs; 0.3 µM primer; 0.02 U/µl KAPA HiFi Hot Start DNA Polymerase	
	Nested reaction	
	F: 5'- AAATGTAAGCGGCCATGTTTC -3'<br>R: 5'- CTACTTCTGTCTCTGTTTGTGGT-3'	
	95°C 3 min (1x); 98°C 20 sec, 59°C 15 sec, 72°C 30 sec (35x); 72°C 1 min (1x); 10°C ∞	
	1X KAPA HiFi buffer; 0.3 mM dNTPs; 0.3 µM primer; 0.02 U/µl KAPA HiFi Hot Start DNA Polymerase	
pmtrap	*Preamplification*	
	F: 5'- GGAAGTATAGGAGAAGAAAACT-3'<br>R: 5'- TCTTCTGGTAGTTTGAATTGGT -3'	(Srisutham et al., 2018)
	95°C 3 min (1x); 98°C 20 sec, 60°C 15 sec, 72°C 60 sec (30x); 72°C 1 min (1x); 10°C ∞	This study
	1X KAPA HiFi buffer; 0.3 mM dNTPs; 0.3 µM primer; 0.02 U/µl KAPA HiFi Hot Start DNA Polymerase	
	Nested reaction	
	F: 5'- GCACCTGTTCTCCCTCCAAA-3'<br>R: 5'- TTGAGGTCGTTCCTCCAGTC -3'	
	95°C 3 min (1x); 98°C 20 sec, 66°C 15 sec, 72°C 25 sec (25x); 72°C 1 min (1x); 10°C ∞	
	1X KAPA HiFi buffer; 0.3 mM dNTPs; 0.3 µM primer; 0.02 U/µl KAPA HiFi Hot Start DNA Polymerase	
	1X KAPA HiFi buffer; 0.3 mM dNTPs; 0.3 µM primer; 0.02 U/µl KAPA HiFi Hot Start DNA Polymerase	

**Target region**	**PCR Primer sequence and cycling conditions**	**Reference**
Pm02	*Preamplification*	
	F: 5’- CGAATTCTATGGACACATGG -3’<br>R: 5’- AAAAGAGGAGAAGGCGATCA -3’	This study
	95°C 3 min (1x); 98°C 20 sec, 65°C 15 sec, 72°C 20 sec (20x); 72°C 2 min (1x); 10°C ∞	
	1X KAPA HiFi buffer; 0.3 mM dNTPs; 0.3 µM primer; 0.02 U/µl KAPA HiFi Hot Start DNA Polymerase	
	Nested reaction	
	F: 5’- GGGGCATAAAGGAAAAAC -3’<br>R: 5’- GAATTTTTGAATAACAAGAAACC-3’	(Bruce et al., 2006)
	95°C 3 min (1x); 98°C 20 sec, 57°C 15 sec, 72°C 15 sec (35x); 72°C 1 min (1x); 10°C ∞	This study
	1X KAPA HiFi buffer; 0.3 mM dNTPs; 0.3 µM primer; 0.02 U/µl KAPA HiFi Hot Start DNA Polymerase	
Pm09	*Preamplification*	
	F: 5’- ACAACATTGTTAAAGGAGTT -3’*<br>R: 5’- GTTCATAACTTTGATCTTAAC -3’**	*This study; **(Bruce et al., 2006)
	95°C 3 min (1x); 98°C 20 sec, 58°C 15 sec, 72°C 30 sec (25x); 72°C 1 min (1x); 10°C ∞	This study
	1X KAPA HiFi buffer; 0.3 mM dNTPs; 0.3 µM primer; 0.02 U/µl KAPA HiFi Hot Start DNA Polymerase	
	Nested reaction	
	F: 5’- ACGATAATAATATAAATGGGG-3’<br>R: 5’- CATTTGACCAATTTAACACATTC -3’	(Bruce et al., 2006)
	95°C 3 min (1x); 98°C 20 sec, 60°C 15 sec, 72°C 20 sec (40x); 72°C 1 min (1x); 10°C ∞	This study
	1X KAPA HiFi buffer; 0.3 mM dNTPs; 0.3 µM primer; 0.02 U/µl KAPA HiFi Hot Start DNA Polymerase	

Supplementary Table S2. QIAxcel running conditions used for indel-based genotyping analysis.

Marker region	QIAxcel cartridge	Resolution	Method	Alignment marker	Size marker	Size marker concentration
*pmmsp1*F2	High Resolution	20 bp	0M400	15 – 1000 bp	50 – 800 bp	30 ng/µl
pmtrap	High Resolution	3-5 bp	0M800	15 – 600 bp	25 - 500 bp	30 ng/µl
Pm02	High Resolution	3-5 bp	0M800	15 – 600 bp	25 - 500 bp	30 ng/µl
Pm09	High Resolution	3-5 bp	0M800	15 – 600 bp	25 - 500 bp	30 ng/µl

Supplementary Table S3. Number of different genotypes detected for samples i.01 to i.21 in each of the 15 timepoints for both marker *pmtrap* and *pmmsp1* F2. If the number of alleles for both markers correspond, the numbers are marked in grey.

ID	marker	Timepoints														
		SCR	H8	H16	H24	H32	H40	H48	H56	H64	H72	H96	H120	D6	D7	D14
i.01	*pmtrap*	1	1	1	1	1	1	1	1	1	1	1	1	1	1	
	pmmsp1 F2	1	1	1	1	1	1	1	1	1	1	1	1	1	1	
i.02	*pmtrap*	6	8	5	6	1	8	7	8	8	8	8	6	8	8	
	pmmsp1 F2	1	2	2	1		2	2	2	2	2	2	2	3	2	
i.03	*pmtrap*	5	5	3	4	5	5	6	5	4	6	5	3	6	3	
	pmmsp1 F2	2	2	1	2	1	2	1	1	1	1	2	2	1	2	
i.04	*pmtrap*	2	2	4	4	5	1	1	3	2	2	4	5	3	6	1
	pmmsp1 F2	1	1	1	1	1	1	1	1	1	1	1	1	1	1	
i.05	*pmtrap*	7	7	7	7	6	6	6	7	6	7	7	5	6	5	
	pmmsp1 F2	2	1	1	2	1	1	1	1	1	1	1	1	1	1	
i.06	*pmtrap*	2	1	1	3	4	4	4	4	4	4	4	3	4	3	
	pmmsp1 F2	2	2	2	2	2	2	2	2	2	2	2	2	2	1	
i.07	*pmtrap*	1	1	4	4	1	1	1	1	1	3	2	4	2	2	
	pmmsp1 F2	1	1	1	1	1	1	1	1		1	1	1			
i.08	*pmtrap*	1	1	1	1	1	1	1	1	1	1	1	1	1	1	
	pmmsp1 F2	1	1	1	1	1	1	1	1	1	1	2	1	1	1	
i.09	*pmtrap*	2	3	3	3	3	3	3	3		3	4	3		4	
	pmmsp1 F2	1	1	1	2	2	2	2	1		2	2	2		1	
i.10	*pmtrap*	1	1	0	1	1	1	1	1	1	4	1	1			
	pmmsp1 F2	2	2	1	1	1	1	1	1	1	2	2	1	1	1	
i.11	*pmtrap*	1	1	1	1	1	1	1	1	4	1	1	1	1	1	
	pmmsp1 F2	1	1	1	1	1	1	1	1	1	1	1	1	1	1	
i.12	*pmtrap*	3	3	3	3	3	3	1	3	2	1	3	3	3	3	
	pmmsp1 F2	2	2	2	2	2	2	2	2	2	2	2	2	2	2	
i.13	*pmtrap*	4	5	1	5	4	2	4	5	5	3	4	5	3	5	
	pmmsp1 F2	2	2	1	2	2	2	2	2	2	3	1	2	2	2	
i.14	*pmtrap*	1	1	1	1	1	1	1	1	1	1	1	1	1	1	1
	pmmsp1 F2	1	1	1	1	1	1	1	1	1	1	1	1	1	1	1
i.15	*pmtrap*	1	1	1	1	1	1	1	1	1		1	1		1	2
	pmmsp1 F2	1	1	1	1	1	1	1	1	1	1	1		1		2
i.16	*pmtrap*	1	1	1	1	1	1	1	1	1	1	1	1	1	1	
	pmmsp1 F2															
i.17	*pmtrap*	1	1	1			1	1	4	1	4	1	1		1	
	pmmsp1 F2															

ID	marker	Timepoints														
		**SCR**	**H8**	**H16**	**H24**	**H32**	**H40**	**H48**	**H56**	**H64**	**H72**	**H96**	**H120**	**D6**	**D7**	**D14**
**i.18**	***pmtrap***	**5**	**4**	**4**	**4**	**4**	**5**	**4**	**4**	**4**	**1**	**4**	**2**	**3**	**3**	
	***pmmsp1* F2**															
**i.19**	***pmtrap***	**3**	**3**	**3**		**3**			**1**	**1**		**1**	**3**	**1**	**1**	
	***pmmsp1* F2**															
**i.20**	***pmtrap***	**1**	**1**	**1**	**1**	**1**	**1**	**1**	**1**	**1**	**1**	**1**	**1**	**2**	**2**	**2**
	***pmmsp1* F2**															
**i.21**	***pmtrap***															
	***pmmsp1* F2**	**2**	**2**	**2**	**2**	**2**		**1**	**2**	**2**	**2**	**2**	**2**	**2**		

Supplementary Table S4. Mock dilutions of two samples of different sizes: sample 1 (small allele, 295 bp) and sample 2 (big allele, 479 bp) were mixed with one another at different concentrations as noted in the table. In more detail, from each sample six dilutions were generated using the stock from the respective other sample as a diluent. All dilutions were measured using automated capillary electrophoresis with the QIAxcel system. As an output of this, size and concentration are given. NA = not applicable. Neg = negative

Dilutions	Results Sample 1		Results Sample 2	
	Size [bp]	Concentration [ng/µl]	Size [bp]	Concentration [ng/µl]
Sample 1	295	16,49	NA	NA
Sample 2	NA	NA	479	17,49
Sample 1 : Sample 2	295	7,72	482	5,38
Sample 1 : Sample 2 (1:10)	296	8,23	484	4,98
Sample 1 : Sample 2 (1:100)	297	3,7	481	12,84
Sample 1 : Sample 2 (1:1000)	296	7,76	484	3,79
Sample 1 : Sample 2 (1:10 000)	295	11,81	483	2,77
Sample 1 : Sample 2 (1:100 000)	294	19,22	neg	neg
Sample 2 : Sample 1 (1:10)	295	3,82	481	10
Sample 2 : Sample 1 (1:100)	295	1,35	480	10,28
Sample 2 : Sample 1 (1:1000)	296	1,14	478	16,19
Sample 2 : Sample 1 (1:10 000)	295	1,27	478	14,94
Sample 2 : Sample 1 (1:100 000)	neg	neg	477	18,08

Supplementary Table S5. Validation of amplicon lengths from automated capillary gel electrophoresis via Sanger sequencing. Measured amplicon length of 10 samples A-J is compared between size measures from Sanger sequencing (“Sanger”) to automated capillary gel electrophoresis (“QIA”). Divergence (“Div.”) is given in the third column for each marker. REF: References used are LT594633 for Pm02 and *pmtrap*, LT594622 for Pm09, LT594628 for *pmmsp1* F2.

	**Pm02**			**Pm09**			***pmmsp1* F2**			***pmtrap***		
	**Sanger [bp]**	**QIA [bp]**	**Div. [bp]**	**Sanger [bp]**	**QIA [bp]**	**Div. [bp]**	**Sanger [bp]**	**QIA [bp]**	**Div. [bp]**	**Sanger [bp]**	**QIA [bp]**	**Div. [bp]**
A	201	202	1	335	331	4	302	298	4	432	427	5
B	213	213	0	318	320	2	482	488	6	372	373	1
C	201	201	0	335	332	3	302	295	7	432	426	6
D	213	212	1	335	332	3	497	476	21	372	370	2
E	217	221	4	318	315	3	479	483	4	372	369	3
F	205	200	5	335	332	3	302	310	8	432	427	5
G	209	219	10	335	331	4	503	497	6	372	369	3
H	201	200	1	335	331	4	302	310	8	432	427	5
I	209	219	10	335	329	6	497	492	5	396	391	5
J	209	220	11	318	314	4	302	296	6	372	369	3
REF	213			318			627			408		

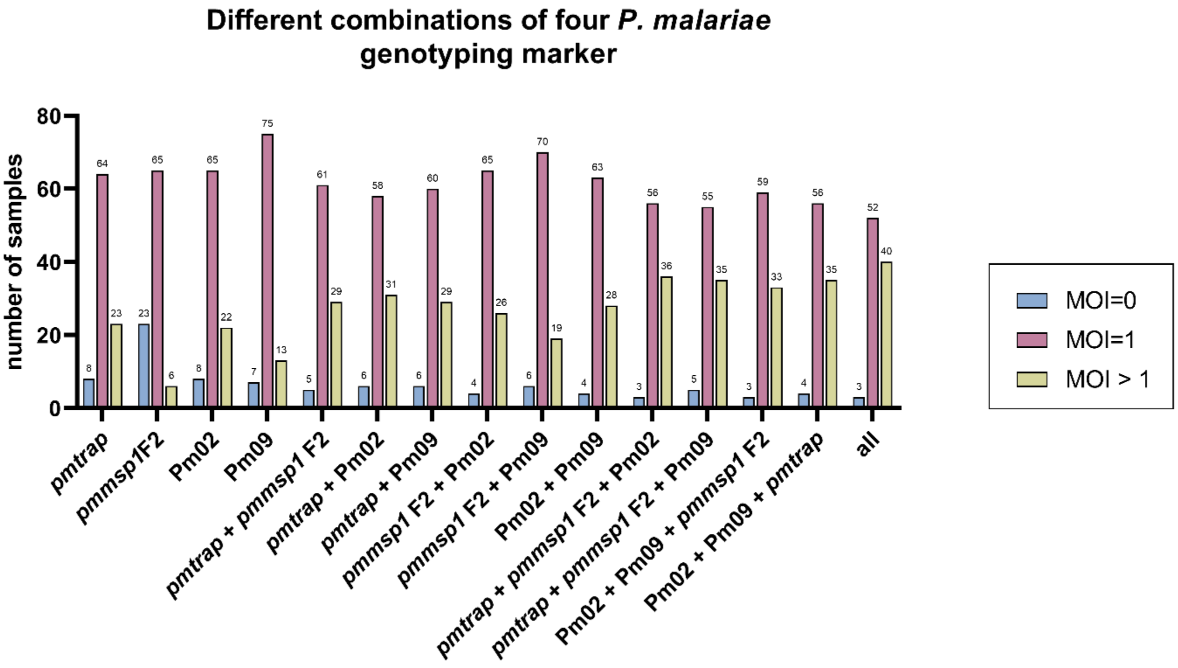

Supplementary Figure S1. Comparison of a single marker to different combinations of the four *P. malariae* marker Pm02, Pm09, *pmmsp1* F2 and *pmtrap* regarding the capability of displaying the highest genetic diversity. For each combination the number of samples with a multiplicity of infection (MOI) of 0 is given in blue, i.e. the number of PCR negative samples for the respective marker or combination. For the number of samples with an MOI of 1 in red and in yellow for an MOI >1. In total 11 possible combinations are shown, six combinations out of two markers, four possible combinations out of three markers included, and one combination including all four markers.

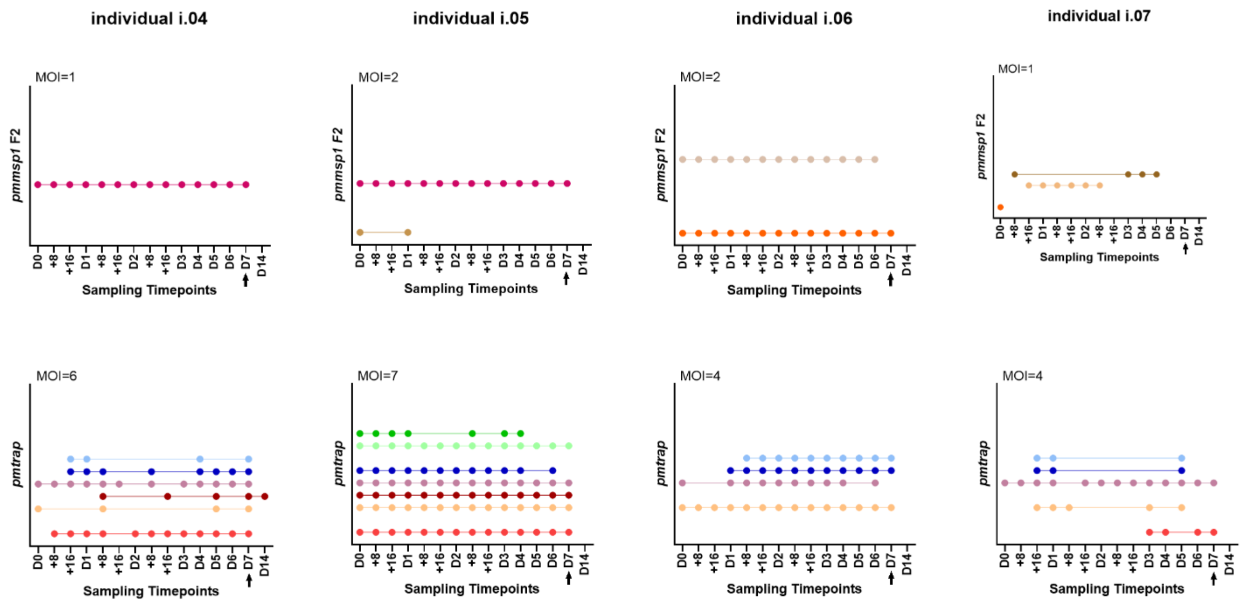
individual i.04
individual i.05
individual i.06
individual i.07
MOI=1
MOI=2
MOI=2
MOI=1
MOI=6
MOI=7
MOI=4
MOI=4
pmmsp1 F2
pmtrap
D0 +8 +16 D1 +8 +16 D2 +8 +16 D3 D4 D5 D6 D7 D14
Sampling Timepoints

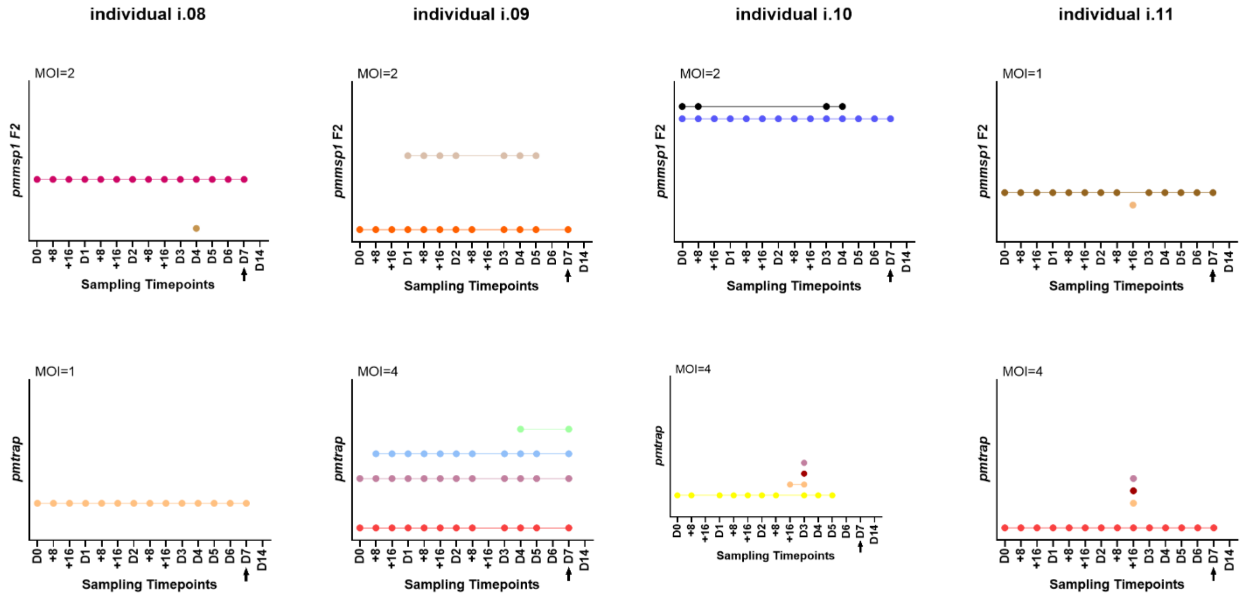
individual i.08
individual i.09
individual i.10
individual i.11
MOI=2
MOI=2
MOI=2
MOI=1
MOI=1
MOI=4
MOI=4
MOI=4
pmmsp1 F2
pmtrap
D0 +8 +16 D1 +8 +16 D2 +8 +16 D3 D4 D5 D6 D7 D14
Sampling Timepoints

individual i.12

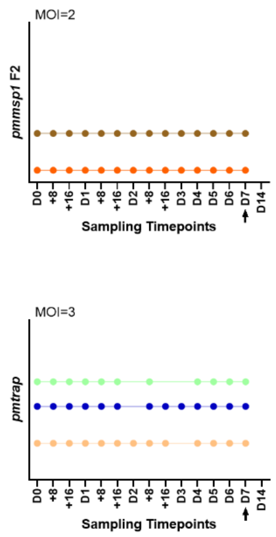

individual i.13

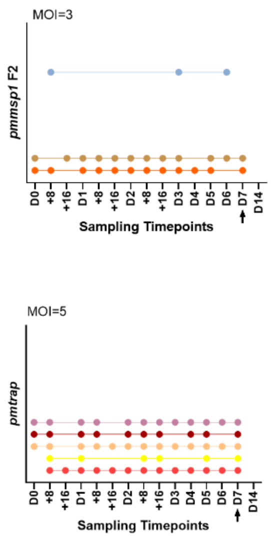

individual i.14

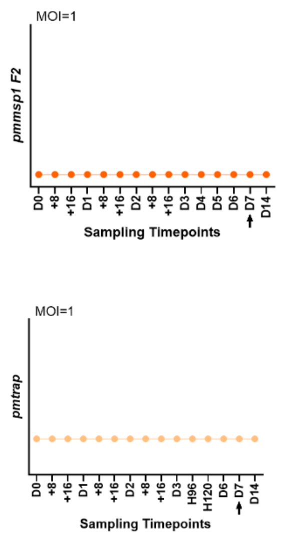

individual i.15

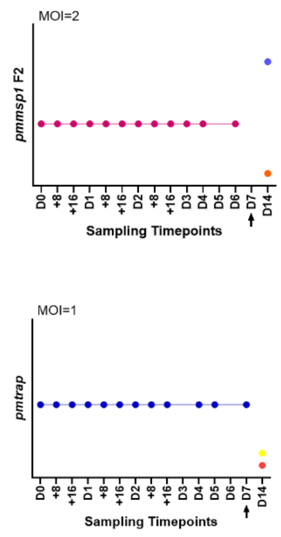

individual i.16

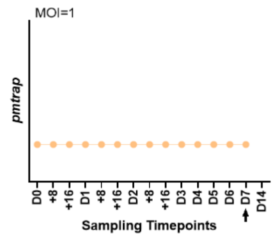

individual i.17

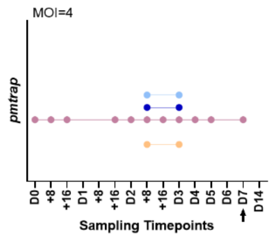

individual i.18

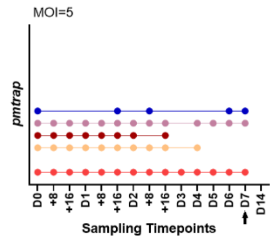

individual i.19

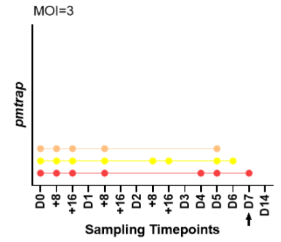

individual i.20

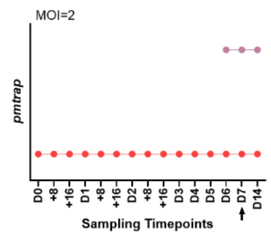

individual i.21

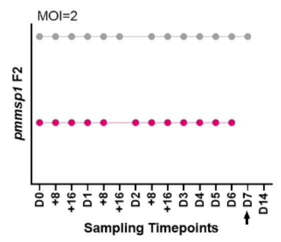

Supplementary Figure S2. Genotypes detected in 18 individuals using markers *pmmsp1* F2 (upper row) and *pmtrap* (lower row). Different colours represent different genotypes for each marker. The arrow indicates treatment with artemether-lumefantrine on day 7. For individual i.04 – i.15 data is available for both markers. However, for individual i.16 – i.20 data is available for *pmtrap* and for individual i.21 for *pmmsp1* F2 only. The respective other marker was negative in these 6 cases. MOI = multiplicity of infection, highest number if genotypes detected among all 15 timepoints.

References

BRUCE, C., M., MACHESO, A., GALINSKI, R., M. & BARNWELL, W., J. 2006. Characterization and application of multiple genetic markers for<i>Plasmodium malariae</i>. *Parasitology,* 134**,** 637-650.

EKOKA MBASSI, D., MOMBO-NGOMA, G., HELD, J., OKWU, D. G., NDZEBE-NDOUMBA, W., KALKMAN, L. C., EKOKA MBASSI, F. A., PESSANHA DE CARVALHO, L., INOUE, J., AKINOSHO, M. A., DIMESSA MBADINGA, L. B., YOVO, E. K., MORDMÜLLER, B., KREMSNER, P. G., ADEGNIKA, A. A., RAMHARTER, M. & ZOLEKO-MANEGO, R. 2023. Efficacy and safety of ivermectin for the treatment of Plasmodium falciparum infections in asymptomatic male and female Gabonese adults - a pilot randomized, double-blind, placebo-controlled single-centre phase Ib/IIa clinical trial. *EBioMedicine,* 97**,** 104814.

GROGER, M., VELETZKY, L., LALREMRUATA, A., CATTANEO, C., MISCHLINGER, J., ZOLEKO-MANEGO, R., ENDAMNE, L., KLICPERA, A., KIM, J., NGUYEN, T., FLOHR, L., REMPPIS, J., MATSIEGUI, P.-B., ADEGNIKA, A. A., AGNANDJI, S. T., KREMSNER, P. G., MORDMÜLLER, B., MOMBO-NGOMA, G. & RAMHARTER, M. 2018. Prospective Clinical Trial Assessing Species-Specific Efficacy of Artemether-Lumefantrine for the Treatment of Plasmodium malariae, Plasmodium ovale, and Mixed Plasmodium Malaria in Gabon. *Antimicrobial Agents and Chemotherapy,* 62**,** AAC.01758-17.

GUIMARÃES, L. O., WUNDERLICH, G., ALVES, J. M. P., BUENO, M. G., RÖHE, F., CATÃO-DIAS, J. L., NEVES, A., MALAFRONTE, R. S., CURADO, I., DOMINGUES, W. & KIRCHGATTER, K. 2015. Merozoite surface protein-1 genetic diversity in Plasmodium malariae and Plasmodium brasilianum from Brazil. *BMC Infectious Diseases,* 15.

MANEGO, R. Z., MOMBO-NGOMA, G., WITTE, M., HELD, J., GMEINER, M., GEBRU, T., TAZEMDA, B., MISCHLINGER, J., GROGER, M., LELL, B., ADEGNIKA, A. A., AGNANDJI, S. T., KREMSNER, P. G., MORDMÜLLER, B., RAMHARTER, M. & MATSIEGUI, P. B. 2017. Demography, maternal health and the epidemiology of malaria and other major infectious diseases in the rural department Tsamba-Magotsi, Ngounie Province, in central African Gabon. *BMC Public Health,* 17**,** 130.

SRISUTHAM, S., SARALAMBA, N., SRIPRAWAT, K., MAYXAY, M., SMITHUIS, F., NOSTEN, F., PUKRITTAYAKAMEE, S., DAY, N. P. J., DONDORP, A. M. & IMWONG, M. 2018. Genetic diversity of three surface protein genes in Plasmodium malariae from three Asian countries. *Malaria Journal,* 17.

WALSH, P. S., ERLICH, H. A. & HIGUCHI, R. 1992. Preferential PCR amplification of alleles: mechanisms and solutions. *PCR Methods Appl,* 1**,** 241-50.

WOLDEAREGAI, T. G., LALREMRUATA, A., NGUYEN, T. T., GMEINER, M., VELETZKY, L., TAZEMDA-KUITSOUC, G. B., MATSIEGUI, P. B., MORDMÜLLER, B. & HELD, J. 2019. Characterization of Plasmodium infections among inhabitants of rural areas in Gabon. *Sci Rep,* 9**,** 9784.
